# A Rapid, High-Throughput Method for the Construction of Mutagenesis Libraries

**DOI:** 10.3390/biom15111511

**Published:** 2025-10-25

**Authors:** Yuxin Lu, Shuting Meng, Xinyi Guan, Pengying He, Dongxin Zhao

**Affiliations:** 1 School of Chinese Materia Medica, Nanjing University of Chinese Medicine, Nanjing 210023, China; 2 Shanghai Institute of Materia Medica, Chinese Academy of Sciences, Shanghai 210203, China; 3 School of Pharmacy, Henan University, Kaifeng 475004, China; 4 University of Chinese Academy of Sciences, Beijing 100049, China

**Keywords:** high-throughput, library preparation, sequencing, normalization, oligo-directed mutagenesis

## Abstract

As synthetic biology advances toward precise design, the construction of high-quality mutant libraries has become essential for large-scale functional screening. Traditional approaches, such as random and saturation mutagenesis, often suffer from low accuracy, high bias, and limited coverage. An ideal method should offer controlled mutagenesis, comprehensive coverage, high throughput, operational simplicity, and controllable outcomes, enabling effective large-scale screening. Here, we developed a high-throughput, precisely controlled method for constructing a mutagenesis library based on chip-based oligonucleotide synthesis. Using *PSMD10* as a model, we constructed a full-length amber codon scanning mutagenesis library with 93.75% mutation coverage. Among the five polymerases evaluated, KAPA HiFi HotStart, Platinum SuperFi II and Hot-Start Pfu DNA Polymerase demonstrated higher amplification efficiency and lower chimera formation rates, making them preferred enzymes for optimized library construction. Analysis of unmapped reads highlighted key technical factors, such as oligonucleotide synthesis errors and chimeric sequence formation caused by incomplete extension of DNA polymerase or synthesis across discontinuous templates during PCR. To improve efficiency and fidelity, we recommend refining PCR conditions and strengthening oligo synthesis quality control. We establish an efficient, scalable, precisely controlled mutagenesis library construction strategy tailored for high-throughput functional research and recommend using a high-fidelity, low-bias polymerase to ensure quality.

## 1. Introduction

As synthetic biology and protein engineering enter the era of precision design, systematic elucidation of functional motifs in biomacromolecules has emerged as a key strategy combining directed evolution [1] and rational design paradigms. The rapid development of high-throughput sequencing technology [2,3] and the rise in synthetic biology [4] have made the construction of high-quality mutation libraries a critical component for achieving high-throughput functional screening. An optimal mutagenesis library should have high mutation coverage, diverse mutation profiles, and uniform variant distribution for deep functional phenotyping.

Conventional approaches to gene mutation research primarily rely on random mutagenesis and saturation mutagenesis. Random mutagenesis is a simple strategy for the construction of mutant libraries, as it requires no prior structural or functional information, such as domains or motifs [5]. By introducing extensive random mutations, this technology generates highly diverse variants from a single gene, allowing for the creation of libraries with vast numbers of genetic variants. Even in the absence of detailed structural or functional data, each variant can be isolated and individually assessed for its genotype-phenotype relationship. These genetic variants can be obtained through methods such as random shuffling and error-prone PCR (epPCR). This method, developed by Leung et al. [6], utilizes a low-fidelity DNA polymerase to introduce random mutations during the PCR amplification of a target gene. The resulting mutagenized PCR products are then ligated into plasmid vectors for library construction [7]. However, epPCR introduces mutations by increasing the polymerase error rate, predominantly generating point mutations such as base substitutions, but is inefficient at producing more complex types like insertions or deletions. Consequently, it cannot encompass the full spectrum of genetic variations. Although simple to perform, its low and poorly controlled mutation frequency limits both the diversity and representativeness of the resulting library. Given the low frequency of beneficial mutations and the high incidence of ineffective or deleterious ones, library size must be carefully controlled to mitigate these issues [8]. Furthermore, due to the degeneracy of the genetic code, as well as mutation tendencies introduced by PCR and the inherent characteristics of the employed polymerase, epPCR often exhibits significant mutational preference [9]. 

Saturation mutagenesis is a targeted library creation technique designed to systematically replace the amino acid(s) at one or more specific positions within a gene. This is achieved using synthetic oligonucleotides that contain a randomized codon flanked by wild-type sequences [10]. A key objective in constructing such a library is to ensure representation of all 20 standard amino acid substitutions at each targeted site. In practice, target sites are randomized using degenerate primers, with NNK (where N is A/C/G/T and K is G/T) being a preferred alternative to NNN. While both encode all 20 canonical amino acids, the NNK codon set reduces redundancy from 64 to 32 codons and excludes two of the three stop codons, thereby significantly lowering the screening burden compared to the fully degenerate NNN mixture [11]. However, these conventional degenerate codons still generate libraries with inherent limitations, including residual codon redundancy and uneven amino acid representation. These issues are compounded as the number of targeted sites increases, leading to exponential growth in amino acid bias and making the screening for low-frequency positive clones increasingly challenging. To overcome these limitations, more advanced strategies have been developed. One approach incorporates specialized di- and trinucleotide phosphoramidites during oligonucleotide synthesis to achieve precise codon-level control, but it relies on costly custom reagents and complex workflows [12]. The maximum efficiency (MAX) randomization strategy constructs unbiased and redundancy-free mutant libraries by combining multiple 9-mer primers followed by hybridization, ligation, and PCR amplification. Although this method eliminates the need for sophisticated reagents, its multi-step procedure results in higher costs and greater operational complexity compared to conventional NNN/NNK strategies [13,14].

Design and synthesize the required diversified oligonucleotides, and introduce these oligonucleotides into the specific site of the target gene using PCR technology, providing a new approach to address the aforementioned limitations. High-throughput array-based DNA synthesis enables cost-effective and scalable production of diversified oligonucleotide pools [15], which have been widely applied in synthetic biology and functional genomics, including gRNA library design, barcoding, and regulatory element screening [16,17,18,19]. Over the past years, this technology has been adapted for the construction of deep mutational scanning libraries [20], insertion libraries, and even full-gene and pathway assemblies [21,22].

Here, we present a method for constructing mutagenesis libraries using high-throughput oligonucleotide synthesis technology. Designed sequences carrying diverse mutations are PCR amplified from chemically synthesized oligonucleotides and subsequently assembled into full-length genes via Gibson assembly. An intermediate plasmid vector and high-fidelity DNA polymerase enhance assembly efficiency and sequence accuracy, enabling precise and uniform mutation incorporation. Combined with next-generation sequencing (NGS), this approach allows assessment of library quality and mutation coverage.

Previous studies have demonstrated that introducing an amber codon (TAG) into genes, in conjunction with Genetic Code Expansion Technology (GCET), enables site-specific incorporation of unnatural amino acids (UAAs) bearing functional or bio-reactive groups into target proteins. Under specific conditions, such as UV irradiation or proximity-induced reactions, these UAAs can form covalent bonds with neighboring residues of interacting proteins, allowing in situ capture and covalent crosslinking of protein–protein interactions. This strategy facilitates identification of residues in direct contact with the target protein. For example, Coin et al. used TAG mutagenesis and genetic code expansion to site-specifically incorporate photo-crosslinkable p-Azido-L-phenylalanine (Azi) and clickable Nε-(2-propynyloxycarbonyl)-L-lysine (Ffact) into class B GPCR CRF1R in mammalian cells, enabling in-cell crosslinking with its endogenous ligand Ucn1 under native conditions. This approach captured multiple receptor-ligand contact points and facilitated building a high-resolution full-length CRF1R-Ucn1 complex model [23].

We constructed a full-length amber codon (TAG) scanning mutagenesis library of the *PSMD10* gene, in which each amino acid position was individually mutated to an amber stop codon. NGS analysis demonstrated 93.75% mutation coverage, high mapping efficiency, and minimal dropout variants, confirming method robustness. Unmatched sequences were primarily caused by the formation of chimeric sequences due to incomplete extension by DNA polymerase or DNA synthesis across discontinuous templates during PCR, as well as oligonucleotide synthesis errors [24]. Our systematic evaluation of five high-fidelity DNA polymerases revealed that KAPA HiFi HotStart DNA Polymerase, Platinum SuperFi II DNA Polymerase and Hot-Start Pfu DNA Polymerase exhibited superior performance in both construction efficiency and chimera formation rate. Based on these findings, we recommend using high-fidelity, low-bias polymerase with optimized PCR conditions to ensure both accuracy and efficiency in library construction. This library enables comprehensive identification of PSMD10-interacting proteins and potential crosslinking sites in live cells, offering new opportunities to elucidate its interaction network and functional domains.

## 2. Materials and Methods

### 2.1. Library Design

The coding sequence (CDS) of *PSMD10* (678 bp, encoding 226 amino acids) was obtained from the NCBI RefSeq database (Gene ID: 5716). The sequence was divided into ten sub-libraries (P1–P10), with P1–P9 each containing 24 amino acids (72 bp) and P10 containing the last 9 amino acids of *PSMD10* fused to an additional 15 amino acids derived from the plasmid vector at its C-terminus. Within each sub-library, oligonucleotides were designed with 16–19 bp overlapping homologous arms at both ends for recombination. Each sub-library consists of 24 oligonucleotides, enabling TAG mutations at every amino acid position of *PSMD10*. All designed oligonucleotides were synthesized by GenScript (Piscataway, NJ, USA), with complete sequences provided in Supplementary Table S1. The variant oligonucleotide library was commercially synthesized as a custom GenTitan™ Oligo Pool by GenScript (Piscataway, NJ, USA) using oligonucleotide pool synthesis technology. This proprietary platform employs high-density Complementary Metal Oxide Semiconductor (CMOS) technology combined with microelectrode control to enable high-precision, parallel synthesis on a single chip. The synthesized products were subsequently amplified and purified, then pooled in a lyophilized format and delivered in a single tube, ready for direct use in downstream cloning and other experimental applications.

During the construction of the original plasmid, the vector pQTEV-*PSMD10* (#31332, Addgene, Cambridge, MA, USA) was used as the template to amplify the target sequence using the following primers: EF1α-PSMD10-F: 5′-AGGTAGCTGATATCGAATTCCTGGGGATCCACCGGTCCACGGGGGTGTGTCTAAC-3′ and EF1α-PSMD10-R: 5′-AGGTCAGGCTCGCTGAATTCCCCAATGTCAAGC-3′. The 50 μL PCR reaction mixture contained 25 µL of KAPA HiFi HotStart DNA Polymerase (07958927001, Roche, Basel, Switzerland), 1.5 µL of each 10 µM primer, 10 ng of template plasmid, and nuclease-free water to a final volume of 50 µL. Cycling conditions were: 1 cycle at 98 °C for 30 s; 30 cycles of 98 °C for 20 s, 65 °C for 10 s, and 72 °C for 40 s; followed by a final extension at 72 °C for 1 min. PCR products were analyzed and purified under the following conditions: separation by electrophoresis on a 1% agarose gel (120 V, 35 min); purification using VAHTS DNA Clean Beads; followed by elution in 15 µL of sterile water. Restriction digestion was performed to prepare the plasmid backbone. The 50 µL reaction mixture contained 3 µg of the initial plasmid (EF1α-X-HA-V5-P2A-Hygro), 1 µL EcoRI-HF (R3101V, NEB, Ipswich, MA, USA) restriction enzyme, 5 µL of 10× CutSmart buffer (B7204, NEB, Ipswich, MA, USA), and sterile water to make up the final volume. After incubation at 37 °C for 3 h, the resulting products were analyzed and purified as previously described.

After purification, the backbone and amplified fragments were recombined using Gibson Assembly. The 10 µL reaction mixture contained 5 µL of 2× MultiF SeamLess Assembly Mix (RK21020, ABconal, Wuhan, China), 100 ng of the backbone fragment, 70 ng of the amplified insert, and nuclease-free water to volume. The reaction was incubated at 50 °C for 30 min. The recombinant product was mixed with chemically competent cells by gently mixing the tube, followed by incubation on ice for 30 min. Heat-shock the cells at 42 °C for 45 s, immediately transfer them to an ice bath for 3 min, and add 500 µL of antibiotic-free LB medium. Recover the cells by shaking at 37 °C and 200 rpm for 1 h. After recovery, the culture was spread evenly onto an LB agar plate containing ampicillin and incubated overnight at 37 °C. Individual colonies were picked for Sanger sequencing with T7 primers to verify the correct plasmid. Finally, the original plasmid was extracted using the FastPure EndoFree Plasmid Midi Kit (DC205, Vazyme, Nanjing, China).

### 2.2. Construction of Intermediate Plasmid

Using the preparation of the p1 library as an example, we employed a two-step method to construct an amber codon scanning mutagenesis library for *PSMD10*. The intermediate plasmid (EF1α-*PSMD10*-p1-mid-HA-V5-P2A-Hygro) was constructed in the first step. We began by preparing the plasmid backbone through double-restriction digestion. The 50 µL reaction mixture contained 3 µg of the initial plasmid (EF1α-*PSMD10*-HA-V5-P2A-Hygro), 1 µL each of XhoI-HF (R0146V, NEB, Ipswich, MA, USA) and EcoRI-HF restriction enzymes, 5 µL of 10× CutSmart buffer (B6004V, NEB, Ipswich, MA, USA), and sterile water to make up the final volume. Following incubation at 37 °C for 3 h, the products were analyzed by electrophoresis. This enzymatic digestion linearized the plasmid backbone, creating standardized cloning sites for subsequent insertion of mutant library fragments. The products were then purified. The insert fragment for Gibson assembly cloning was PCR-amplified from the original plasmid (EF1α-*PSMD10*-HA-V5-P2A-Hygro) using two primer pairs: PSMD10-scan-up-F: 5′-CGCCGTCCAGGCACCTCGATTAGTTCTCGAG-3′ with PSMD10-Rgn1-up-R: 5′-CCATGGTGGCGACCGGTGGATCCCCCGGGC-3′, and PSMD10-Rgn1-dn-F: 5′-GCCCGGGGGATCCACCGGTCGCCACCATGGGAGACGTAGGGATAACAGGGTAATCGTCTCTATTCTGGCCGATAAATCCCTGGCTACTAGA-3′ with PSMD10-scan-dn-R: 5′-GAGATGCAATAGGTCAGGCTCTCGCTGAATTC-3′. The 50 µL PCR reaction mixture contained 25 µL of KAPA HiFi HotStart DNA Polymerase, 1.5 µL of each 10 µM primer, 10 ng of template plasmid, and nuclease-free water to a final volume of 50 µL. Cycling conditions were: 1 cycle at 98 °C for 30 s; 23 cycles of 98 °C for 20 s, 65 °C for 10 s, and 72 °C for 60 s; followed by a final elongation at 72 °C for 1 min. The products were then subjected to the same analysis and purification procedure.

Ligation, transformation, selection of individual clones for Sanger sequencing, and plasmid extraction were all performed using the same methods as for the original plasmid construction.

### 2.3. Construction of a PSMD10 Full-Gene Amber Codon Scanning Mutagenesis Library

Following the construction of the intermediate plasmid, the P1 sub-library was generated. First, the appropriate number of PCR amplification cycles was determined. By setting up a gradient of cycle numbers, the amplification efficiency and product specificity under different cycle conditions were compared to determine suitable amplification parameters. After analyzing the amplification products via agarose gel electrophoresis, the cycle number that produced clear bands with moderate intensity and no nonspecific amplification was selected as the preferred cycle number for subsequent experiments. The plasmid backbone was linearized by restriction digestion under the following conditions: 3 µg of intermediate plasmid (EF1α-*PSMD10*-p1-mid-HA-V5-P2A-Hygro), 1 µL of BsmBI-v2 (R0739S, NEB, Ipswich, MA, USA), 1 µL of EcoRI-HF, 5 µL of NEBuffer r3.1 (B7030S, NEB, Ipswich, MA, USA), and sterile water to a final volume of 50 µL. The reaction was incubated at 55 °C for 3 h. The PCR products were processed accordingly.

The P1 oligo was amplified by PCR using the synthesized oligo pool as the template, with the following primers, PSMD10-amp-F1: 5′-AGCCCGGGGGATCCACCGGTCGCCCATG-3′ and PSMD10-amp-R1: 5′-TCTAGTAGCCAGGGATTTATCGGCCAGAAT-3′. The 50 μL PCR reaction mixture contained 25 μL of KAPA HiFi HotStart DNA Polymerase, 1.5 μL of each 10 μM primer, 10 ng of template plasmid, and nuclease-free water to a final volume of 50 μL. Cycling conditions: 1 cycle at 95 °C for 3 min, 26 cycles of 98 °C for 20 s, 65 °C for 15 s, 72 °C for 10 s, followed by final elongation at 72 °C for 1 min. Following electrophoretic separation, the target DNA bands were visualized under UV light, followed by purification of the products.

Purified backbone and amplified fragments were subjected to Gibson Assembly. The 10 μL reaction mixture contained 5 μL of 2× MultiF SeamLess Assembly Mix, 100 ng of backbone fragment, 70 ng of amplified insert, and nuclease-free water to volume. After incubation at 50 °C for 30 min, the assembled products were purified using the FastPure Gel DNA Extraction Mini Kit (DC301, Vazyme, Nanjing, China) and eluted in 6 μL nuclease-free water. For transformation, 2 μL of purified product was electroporated into 50 μL of Stbl3 electrocompetent cells. Cells were recovered in 1 mL of antibiotic-free SOC medium at 37 °C with shaking (250 rpm) for 1 h. A 1 mL bacterial culture was serially diluted to prepare 1:10 and 1:100 dilution gradients. Subsequently, 1 mL from each dilution (including the undiluted original culture) was plated onto 15 cm LB agar plates containing 50 µg/mL ampicillin, with three biological replicates prepared for each dilution level. After overnight incubation at 30 °C, colony quantification was performed using OpenCFU software (version 4.0.0, OpenCFU, Cambridge, UK) (threshold set to 0.5; radius detection set to automatic), combined with manual counting to exclude non-colony particles. Individual colonies were randomly selected and verified by Sanger sequencing using T7 primers. For final library construction, the 1:100 dilution was chosen, as it yielded an appropriate number of colonies within a countable range. Finally, bacterial biomass was collected from the corresponding plates, and plasmid DNA was extracted using the FastPure EndoFree Plasmid Midi Kit (DC205, Vazyme, Nanjing, China) to obtain the final P1 sub-library plasmid pool.

### 2.4. Comparative Analysis of High-Fidelity DNA Polymerases

Using the P1 sub-library as a representative, we evaluated five high-fidelity DNA polymerases: Platinum SuperFi II DNA Polymerase (12361010, Thermo Fisher, Waltham, MA, USA), Q5 Hot Start High-Fidelity DNA Polymerase (M0494, NEB, Ipswich, USA), Hot-Start Pfu DNA Polymerase (HM81211, Heavybio, Shenzhen, China), KAPA HiFi HotStart DNA Polymerase (07958927001, Roche, Basel, Switzerland), and TaKaRa Ex Taq DNA Polymerase (RR001Q, TaKaRa, Kusatsu, Japan). Based on the manufacturers’ protocols and the primers’ Tm values, annealing temperatures and cycle numbers were optimized. PCR reactions were then performed under four sets of optimized conditions using a fixed amount (10 ng) of the pre-amplified P1 oligonucleotide library product as the common template, which served as the defined starting material for all experiments to ensure consistency and enable meaningful comparisons across different polymerases. The pre-amplified library product itself was also subjected to NGS, providing a baseline reference for subsequent comparison. All PCR amplification products were validated by NGS, and amplification quality was assessed by comparing target fragment abundance, sequence accuracy, and the proportion of unintended products such as chimeric molecules against the baseline data from the pre-amplified library. Subsequent purification, NGS library preparation, and data analysis were performed as previously described. Detailed reaction components and cycling conditions are provided in Appendix A. Employing a uniform template and baseline sequencing data allowed direct comparison and correlation of PCR results with NGS data, to evaluate polymerase fidelity and performance.

### 2.5. NGS Sequencing Validation

The Illumina NovaSeq platform performs paired-end sequencing (150 bp per end), offering 300 bp of effective coverage per fragment. To maximize sequencing efficiency, we pool 4 or 2 sub-libraries (288 bp or 144 bp total length, respectively) into a single sequencing unit. Unique barcodes are ligated to the target fragments, followed by universal NGS adapter addition, to generate the final sequencing library.

First, perform a first-round PCR amplification using the EF1α-*PSMD10*-p1-HA-V5-P2A-hygro library as a template with primers NGS-P (1-4)-F: 5′-ACACTCTTTCCCTACACGACGCTCTTCCGATCTGAGGGGTGTGTGTCTAACCT-3′ and NGS-P (1-4)-R: 5′-GACTGGAGTTCAGACGTGTGCTCTTCCGATCTGAGCACCTTTTCCCAGAAGG-3′. Thermal cycling conditions: initial denaturation at 95 °C for 3 min; 14 cycles of 98 °C for 20 s, 65 °C for 15 s, and 72 °C for 15 s; final extension at 72 °C for 1 min. Following analysis and purification, the products were quantified using a Qubit™ 4 Fluorometer (Thermo Fisher, Waltham, MA, USA).

For second-round PCR, purified products were amplified with Illumina adapter primers i7: 5′-CAAGCAGAAGACGGCATACGAGATGATCTGGTGACTGGAGTTCAGACGTGTGCTCTTCCGATC-s-T-3′ and i5: 5′-AATGATACGGCGACCACCGAGATCTACACGGCTCTGAACACTCTTTCCCTACACGACGCTCTTCCGATC*T-3′ using the same cycling conditions (8 cycles). Final libraries were validated by electrophoresis, purified, quantified, and prepared for sequencing.

### 2.6. Bioinformatic Analysis

The raw FASTQ files underwent stringent quality control and precise trimming based on predefined flanking sequences for each sub-library, retaining only the target 72 bp regions while filtering out truncated reads to ensure accurate alignment. To ensure a reliable assessment of enzymatic fidelity under varying reaction conditions, for each condition, five independent replicates were generated by randomly extracting 5000 reads from each trimmed FASTQ files. Subsequently, sequencing data were classified according to edit distance and codon features. Through systematic analysis, the following variant types were accurately identified: TAG Mutation, TAG Mutation with 1bp variant, TAG Mutation with additional variants, WT, WT with 1bp variant, WT with additional variants and Multiple TAG events. A strict priority-based classification strategy was employed to ensure each read was uniquely and accurately assigned to its category, preventing duplicate counting. The algorithm integrated the edlib global alignment module to enable efficient computation of edit distances. Finally, all quantitative metrics were consolidated to generate a comprehensive quality assessment, which was visualized using stacked bar plots.

A favorable reaction condition for each enzyme was determined by integrating the mean and standard deviation of TAG Mutation counts across all tested conditions. Based on the selected reaction conditions for each enzyme, NGS data of PCR products-including counts of TAG Mutation, WT, and Multiple TAG-were extracted from five replicate groups. Pairwise statistical comparisons were then conducted to evaluate the significance of differences in enzymatic fidelity among the enzymes.

## 3. Results

We developed a mutagenesis library construction strategy based on high-throughput oligonucleotide synthesis. This method involves designing target mutant sequences and synthesizing corresponding oligonucleotides. All desired mutant sequences are then obtained through a single round of PCR amplification and assembled using an intermediate plasmid. This approach enables controlled mutations across all target gene sites, overcoming the limitations of conventional random mutagenesis methods and ensuring the generation of high-quality libraries with exceptional diversity and accuracy. When coupled with NGS-based quality control, this methodology effectively supports deep mutational scanning, directed evolution, and high-throughput screening applications, demonstrating broad utility across molecular biology research. We established this method and constructed the mutagenesis library using the *PSMD10* gene (which encodes Gankyrin) as an exemplary case. Gankyrin has been described as an oncoprotein that is a component of the 19S regulatory cap of the proteasome and plays crucial roles in the proliferation, invasion, and metastasis of various tumors [25,26]. Gankyrin is overexpressed in various types of tumors, and its high expression predicts tumor progression and poor prognosis in patients [27,28]. These data highlight gankyrin as a potential biomarker in different cancers [29]. Previous studies have shown that *PSMD10* plays its oncogenic role mainly through its ankyrin repeats to mediate protein–protein interactions.

In this study, we constructed a full-length amber codon scanning mutagenesis library for PSMD10, which enables systematic identification of PSMD10-interacting proteins and potential crosslinking sites in live cells [30]. This platform offers new opportunities to explore PSMD10’s interaction network and functional domains, and may help reveal structural features associated with its oncogenic properties.

**Figure 1:**
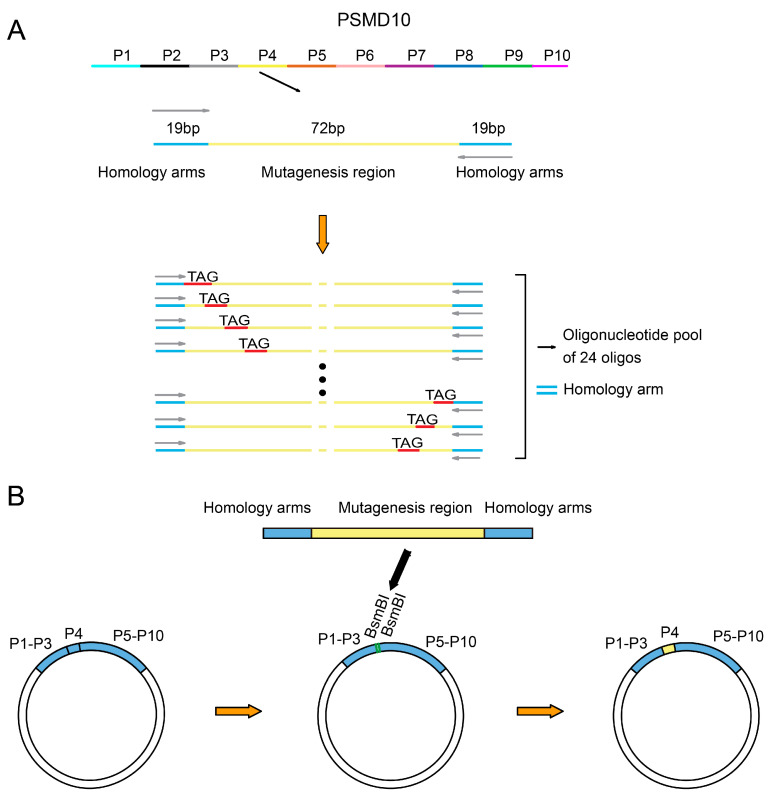
Library design and intermediate plasmid construction strategy. (**A**) Schematic of oligonucleotide library design. The full-length *PSMD10* gene was divided into 10 sublibraries (P1–P10). Taking sublibrary P4 as an example, each sublibrary’s oligonucleotides contain 19 bp constant primer-binding regions (blue segments) at both ends, enabling specific amplification of the corresponding variable region. The central region is the variable segment (yellow), where each position is systematically mutated to an amber stop codon (TAG). Each sublibrary’s oligonucleotide pool consists of 24 unique sequences that share identical primer-binding regions and carry a single TAG mutation at a specific site within the variable region. (**B**) Intermediate plasmid assembly strategy. Using sublibrary P4 as an example, the *PSMD10* gene was divided into constant and variable regions, with the variable region corresponding to each sublibrary. The intermediate plasmid backbone contains predefined BsmBI restriction sites flanking the vacant variable region. During sublibrary construction, all other regions remain unchanged, and only the corresponding variable region sequences are synthesized and inserted into the intermediate plasmid via BsmBI-mediated assembly (indicated by thick black arrows).

Due to inherent limitations of chemical synthesis in producing long oligonucleotides, conventional synthesis methods face challenges including restricted length, exponentially increasing error rates with sequence length, and significantly reduced yield and difficult purification for longer fragments. Additionally, the synthesis of excessively long oligonucleotides incurs substantially higher costs. To address these issues, we first partitioned the full-length *PSMD10* gene (678 bp) into 10 sublibraries (P1–P10), with each of P1–P9 containing 72 bp fragments, while P10 comprised the remaining 27 bp segment plus an additional 45 bp sequence required for vector integration. For each sublibrary, we extended 19 bases at both the 5′ and 3′ ends as primer-binding regions to facilitate specific amplification of the target sequences. Since all variants within a given sublibrary share identical primer-binding sequences, this design ensures high amplification specificity and reproducibility. Notably, the primer-binding regions themselves are mutation-free; all predefined mutations are confined to the central 72 bp variable region, which is subsequently cloned into the mutagenesis library. Each oligonucleotide in the sublibraries carries a single designed TAG (amber codon) mutation, and the synthesized oligonucleotide pool serves as the template for downstream PCR amplification and library assembly (Figure 1A). Additionally, we designed and constructed an intermediate plasmid as a bridging vector by dividing the *PSMD10* gene into constant and variable regions, with the variable region corresponding to each sublibrary to be constructed. The variable region was left vacant in the plasmid and flanked by pre-designed BsmBI restriction sites. During the construction of different sublibraries, only the corresponding variable region sequences needed to be synthesized and inserted into the intermediate plasmid via the BsmBI sites. This strategy not only strictly limits the oligonucleotide synthesis length to within 112 nucleotides to ensure synthesis quality but also reduces the number of fragments required for the final Gibson assembly to two, thereby significantly improving assembly efficiency (Figure 1B).

**Figure 2:**
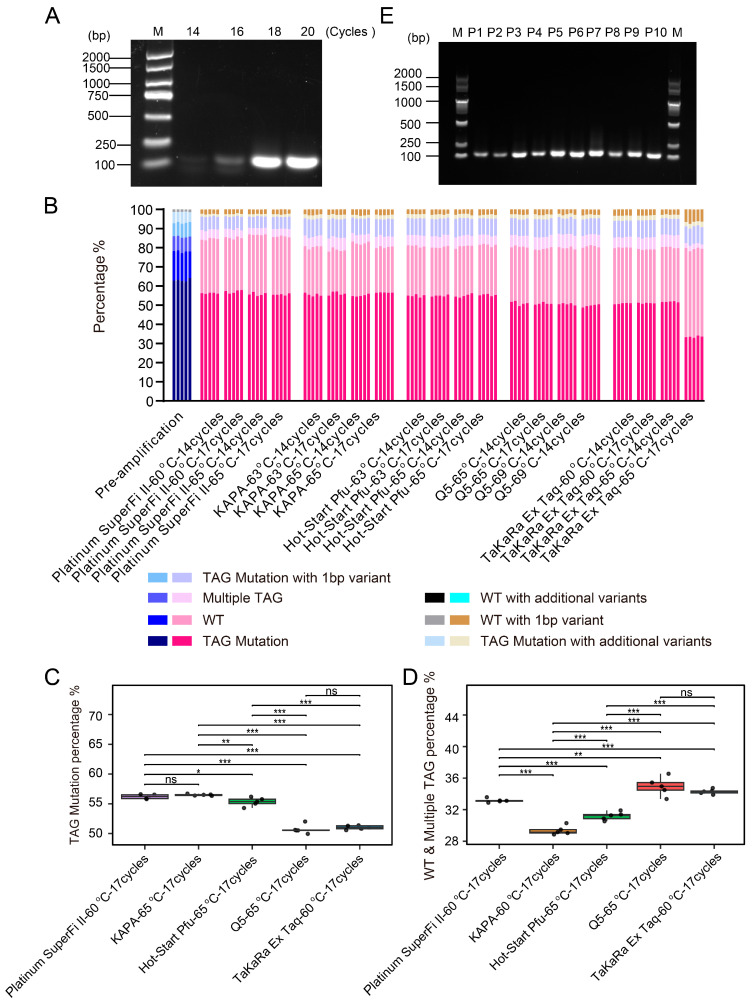
Optimization and validation of PCR amplification conditions for oligonucleotide libraries. (**A**) Effect of different PCR cycle numbers on the amplification efficiency of the P1 sublibrary. PCR was performed using KAPA HiFi HotStart DNA Polymerase at an annealing temperature of 65 °C for 14, 16, 18, and 20 cycles. (**B**) NGS analysis of a common template amplified by five high-fidelity DNA polymerases. Sequencing data were aligned and normalized, and mutation count distribution plots were generated. The X-axis represents the DNA polymerases and their corresponding PCR conditions, while the Y-axis shows the alignment rates of TAG mutant sequences in the PCR products. For each condition, 5000 reads were randomly sampled to form each of five groups for analysis, ensuring representative coverage of the NGS data. The sample labeled “pre-amplification” serves as the pre-amplified P1 oligonucleotide library used as the uniform template and baseline for NGS comparison across all PCR conditions. (**C**,**D**) Comparison of TAG mutation percentage and chimera (wild-type and multiple TAG) percentage across five high-fidelity DNA polymerases under their respective pre-selected PCR conditions. KAPA, Hot-start Pfu, and Q5 were tested at 65 °C, while Platinum SuperFi II and TaKaRa Ex Taq were tested at 60 °C. All reactions were performed using 17 PCR cycles. Boxplots show the TAG mutation and chimera percentages from five replicate groups per enzyme, with each point representing one replicate. * *p* < 0.05, ** *p* < 0.01, *** *p* < 0.001, ns = not significant. (**E**) PCR amplification of the 10 sublibraries was performed under individually optimized conditions using KAPA HiFi HotStart DNA Polymerase with an annealing temperature of 65 °C. Due to differences in primer extension efficiencies, sublibraries P1, P4, P6, and P8 underwent 17 cycles, while P2, P3, P5, P7, P9, and P10 underwent 14 cycles. Agarose gel electrophoresis demonstrated that all sublibraries produced DNA fragments of the expected size. The left lane shows the DNA size marker (unit: bp).

High-fidelity DNA polymerases and optimized PCR reaction conditions contribute to improving the quality of amplified products. Using the P1 sublibrary as a model, we systematically evaluated various DNA polymerases and optimized PCR conditions. Starting with 10 ng of template DNA and an initial annealing temperature of 65 °C based on primer Tm values, gradient PCR was performed using KAPA HiFi HotStart DNA Polymerase with 14, 16, 18, and 20 cycles (Figure 2A). Reactions were harvested at cycles believed to be within the exponential phase to avoid amplification saturation and were analyzed by agarose gel electrophoresis. Preliminary screening was conducted based on the intensity of the target band, amplification specificity, and non-specific background. We evaluated the performance of five high-fidelity DNA polymerases, including Platinum SuperFi II DNA Polymerase, Q5 Hot Start High-Fidelity DNA Polymerase, Hot-Start Pfu DNA Polymerase, KAPA HiFi HotStart DNA Polymerase, and TaKaRa Ex Taq DNA Polymerase. PCR conditions were optimized using different annealing temperatures, taking into account the enzymatic properties and primer melting temperatures (Tm). All PCR reactions were performed using a defined input of a pre-amplified P1 oligonucleotide library. The PCR produces were subject to NGS to assess the accuracy of the sequences. To ensure robustness, we performed random sampling five times and analyzed the TAG sequences. Sequencing reads were classified into the following categories: TAG Mutation (sequences containing the exact TAG mutation), WT (wild-type), Multiple TAG (containing two or more distinct TAG mutations), TAG mutation with 1 bp variant (containing one additional variant besides the target TAG mutation), TAG mutation with additional variants (containing multiple additional variants besides the target TAG mutation), WT with 1 bp variant (overall retaining wild-type features with 1bp variant), and WT with additional variants (overall retaining wild-type features with multiple variants) (Figure 2B).

Next, we quantitatively compared the performance of each polymerase using the annealing temperature that yielded higher average TAG mutation rates under 17 PCR cycles. KAPA HiFi HotStart, Hot-start Pfu, and Q5 Hot Start high-fidelity polymerases performed better at 65 °C, whereas Platinum SuperFi II and TaKaRa Ex Taq exhibited superior performance at 60 °C (Supplementary Table S2). In terms of TAG mutation incorporation efficiency, KAPA, Platinum SuperFi II, and Hot-start Pfu outperformed Q5 and Ex Taq (Figure 2C). Additionally, KAPA polymerase generated the lowest proportion of chimeric reads (Figure 2D). Collectively, these data indicate that KAPA, Platinum SuperFi II, and Hot-start Pfu polymerases not only enable efficient incorporation of TAG mutations, but also maintain a low level of chimeric sequences, including WT carryover and multiple TAG variants, thereby demonstrating higher amplification fidelity and mutation recovery efficiency.

Under a template concentration of 10 ng, robust amplification was obtained using KAPA HiFi DNA Polymerase with an annealing temperature of 65 °C and 17 PCR cycles, yielding approximately 1500 ng of high-quality amplification product. The unintended products, including chimeric molecules, primarily arise from artifacts introduced during the PCR process. For instance, during cycles of incomplete extension, partially extended strands may act as primers in subsequent cycles and anneal to non-template strands, leading to the formation of chimeric molecules composed of two discontinuous template sequences. Furthermore, excessive cycle numbers and suboptimal template quality may also exacerbate the generation of such nonspecific amplification products. In conclusion, rigorously validated high-fidelity, low-bias DNA polymerases under optimized PCR conditions are a preferred choice for library construction, ensuring high accuracy while minimizing PCR-induced artifacts. Using KAPA HiFi HotStart DNA polymerase with an annealing temperature of 65 °C, oligonucleotides from all ten sub-libraries were successfully amplified. Due to differences in primer extension efficiency, sub-libraries P1, P4, P6, and P8 underwent 17 PCR cycles, while P2, P3, P5, P7, P9, and P10 underwent 14 cycles, yielding the expected mutant DNA fragments (Figure 2E).

**Figure 3:**
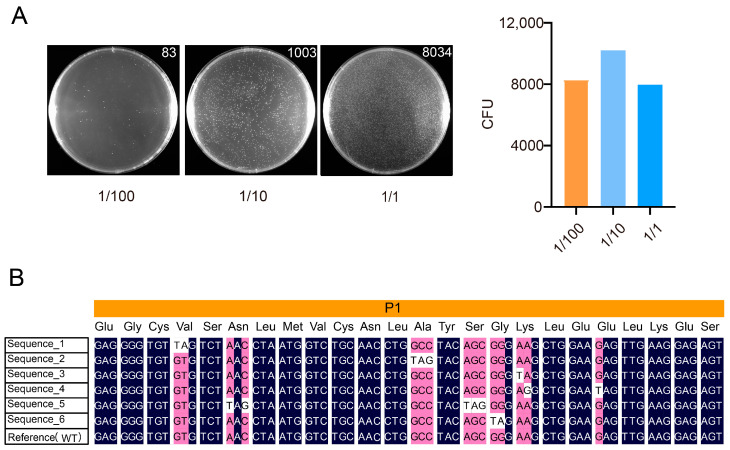
Evaluation and quality validation of the P1 sublibrary construction. (**A**) Colony counts on plates with varying inoculation volumes (selected using 50 µg/mL ampicillin). The left panel shows representative colony images along with their counts. The bar heights represent the normalized average colony counts. The x-axis indicates the different dilution levels. Only the 1/100 dilution data fell within the appropriate countable range and were thus used for library titer calculation. The library titer was calculated as follows: (83 CFU)/(10^−2^ × 1 mL) = 8300 CFU/mL. (**B**) Representative Sanger sequencing results of six randomly selected clones from the P1 sublibrary. Each clone carried the intended mutation, supporting the successful incorporation of designed edits.

For sub-library P1, based on the quantifiable data from the 1/100 dilution series, the final determined library size was approximately 8.3 × 10^3^ CFU (Figure 3A). Six randomly picked clones were sequenced by Sanger sequencing, all of which contained the designed mutations (Figure 3B). The sequences shown are derived from Sanger sequencing chromatograms, as presented in Supplementary Figure S1.

**Figure 4:**
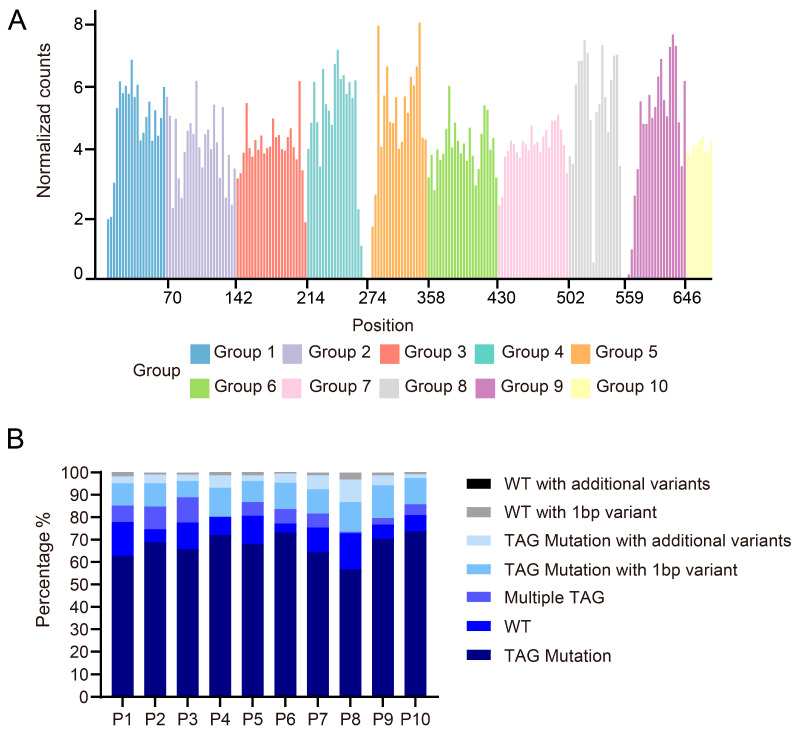
Mutation library sequencing data analysis, normalized mutation count distribution map. The sequencing data underwent alignment and normalization to generate standardized mutation count distribution plots. (**A**) The normalized mutation count distribution bar graph shows the normalized read count distribution corresponding to each tag mutation site in each sub-pool of P1–P10. X-axis: Amino acid position (from start codon); Y-axis: Normalized read counts. (**B**) The bar plot illustrates the distribution of alignment rates for TAG-mutated sequences across all sublibraries (P1–P10), classifying sequencing reads into seven categories (from bottom to top in each column). This quantitative analysis reveals the quality and compositional characteristics of each sublibrary: higher proportions of perfect matches indicate superior library quality, while elevated levels of wild-type or chimeric reads suggest potential anomalies during library construction. These results serve as critical quality control metrics for evaluating library performance and guiding subsequent experimental optimization.

The Illumina NovaSeq platform was employed for 150 bp paired-end sequencing, providing 300 bp of effective coverage per fragment. Four or two sub-libraries (with total lengths of 288 bp or 144 bp, respectively) were pooled for sequencing. Reads were classified into seven categories. A priority-based classification logic prevented duplicate counting, and edit distances were computed using the edlib alignment module. Bioinformatics analysis of the NGS data showed that among 224 pre-designed TAG mutation sites, only 6.25% were undetected, resulting in a high mutation coverage rate of 93.75% (Figure 4A). Through systematic analysis of NGS data from each sub-library, we evaluated the quality characteristics of the mutagenesis library. The results showed that 60–75% of the sequenced reads successfully incorporated the intended TAG stop codon, meeting the expected library construction efficiency. Notably, approximately 11.2% of sequences containing the target TAG mutation exhibited single-base deletions or mutations near the editing site, which most likely originated from errors during oligonucleotide pool synthesis and were subsequently amplified during PCR. Further analysis revealed that although each sequence was designed to contain a single TAG mutation, on average, 5.6% of sequences unexpectedly harbored multiple TAG mutations, while 9.8% retained wild-type sequences without any mutations introduced or existed as a distinct chimeric form (Figure 4B). These phenomena may result from multiple contributing factors, including incomplete extension by DNA polymerase or synthesis across discontinuous templates during PCR amplification caused by the high sequence similarity of DNA templates, suboptimal reaction parameters (such as deviations from the optimal annealing temperature or excessive cycle numbers), and inherent errors in the oligonucleotide synthesis process. The bridge PCR procedure on the Illumina sequencing platform may cause chimeric formations from forward and reverse templates, introducing sequencing artifacts. Furthermore, random errors during PCR or imperfections in oligonucleotide synthesis may result in sequences that largely retain wild-type characteristics but contain one or more base mutations at specific sites. Similarly, the presence of additional base variations in sequences containing the intended TAG mutation underscores the need for stringent quality control of both oligonucleotide synthesis and PCR amplification.

## 4. Discussion

A comprehensive and high-precision mutant library is a key tool for systematically analyzing gene or protein function by introducing predetermined nucleotide variations. An ideal library construction method should provide controlled mutagenesis, comprehensive coverage, high throughput, operational simplicity, and reproducible outcomes, enabling robust application in large-scale, high-throughput functional screening studies. Here, we present a method for constructing mutagenesis libraries using high-throughput oligonucleotide synthesis technology. By employing microarray technology, precisely design and synthesize oligonucleotides with various mutation types and efficiently assemble them into full-length gene sequences, ensuring comprehensive and uniform mutagenesis. All required mutation sequences are obtained through a single PCR reaction, and the pre-designed mutations are efficiently introduced into the library via Gibson assembly, significantly reducing the time required for library construction. By introducing an intermediate plasmid as a transitional vector for library construction, this approach not only circumvents the length limitations of oligonucleotide synthesis but also significantly reduces the number of DNA fragments required for assembly, thereby effectively mitigating the complexity associated with multi-fragment assembly. High-fidelity DNA polymerase is used to ensure the accuracy of the target sequences and minimize the unintended errors. Different mutation types can be designed according to the needs, enabling the construction of various libraries. This method allows precise mutation introduction at specific loci, eliminating randomness and avoiding mutation loss or bias, thereby ensuring the accuracy and uniformity of mutations. It demonstrates broad potential for large-scale mutation screening and high-precision design studies. Based on this, high-throughput NGS is used for deep sequencing analysis of the constructed mutation library, allowing rapid and accurate identification of each mutation site and a comprehensive evaluation of the library’s saturation and uniformity, thereby ensuring the quality and reliability of the library. This method overcomes several limitations of traditional random mutagenesis, including restricted mutation types with substitution bias, imprecise control over mutation frequency, and unintended mutations that disrupt essential terminal sequences or introduce new restriction sites, which compromises the efficiency of correct construct generation and cloning. It also addresses major challenges associated with saturation mutagenesis, such as ineffective mutations caused by degenerate codon redundancy, non-uniform amino acid representation, and the mismatch between exponentially expanding library size and practical screening throughput. By enabling precise control over mutation sites and types, this strategy achieves uniform sequence coverage through a streamlined and efficient workflow, making it particularly suitable for large-scale, high-throughput functional screening studies. Using this method, we constructed an amber codon scanning mutagenesis library for *PSMD10*, achieving defined and uniform mutations at TAG sites. NGS analysis showed that the library reached 93.75% mutation coverage with minimal dropout mutations and high read alignment rates. Through NGS-based validation, we demonstrated that this approach is a robust strategy for building mutagenesis libraries, applicable to protein engineering and genetic code expansion studies.

We evaluated the fidelity and chimera formation rates of five high-fidelity DNA polymerases during PCR amplification. The results showed that KAPA HiFi HotStart DNA Polymerase, Platinum SuperFi II DNA Polymerase and Hot-Start Pfu DNA Polymerase exhibited superior performance compared to Q5 Hot Start High-Fidelity DNA Polymerase and TaKaRa Ex Taq. Furthermore, under a constant template input of 10 ng, both annealing temperature and cycle number significantly influenced product quality. Based on these findings, we recommend prioritizing the use of validated high-fidelity, low-bias polymerases for library construction, along with optimization of reaction conditions to enhance library quality.

We also conducted a systematic analysis of the unintended products. For the predominant TAG mutant chimeras and wild-type sequences, in-depth analysis suggests their formation involves multiple factors: (1) incomplete extension by DNA polymerase or DNA synthesis across discontinuous templates during PCR amplification; (2) the impact of DNA polymerase fidelity; (3) suboptimal settings of PCR reaction parameters (such as annealing temperature and extension time); and (4) inherent technical errors in oligonucleotide pool synthesis. Notably, the single-base mutations and deletions detected in the vicinity of TAG mutation sites further confirm quality control issues during oligonucleotide pool preparation. These findings indicate the necessity to establish more stringent quality control standards for oligonucleotide pool synthesis in subsequent experiments, including but not limited to improving synthesis purity, optimizing purification processes, and enhancing final product quality inspection.

## 5. Conclusions

In summary, we have developed a high-throughput, precisely controlled, and rapid method for constructing mutagenesis libraries. Based on the mutagenesis library construction method developed in this study, we constructed an amber codon scanning mutagenesis library. Systematic detection and analysis of the library using NGS technology confirmed the high reliability and reproducibility of this method. A particularly noteworthy aspect is our in-depth analysis of unmapped sequencing data, through which we identified several key factors affecting data quality. Among these, base mismatches and deletions during oligonucleotide synthesis in microarray preparation were the main technical challenges. Meanwhile, chimeras and nonspecific amplification products generated during high-cycle PCR amplification significantly increased the errors of library construction. Based on the experimental results, we recommend the use of a DNA polymerase with high fidelity and low bias, which offers both excellent accuracy and amplification efficiency, making it well-suited for mutagenesis library construction. To ensure high library quality and reliability, it is essential to optimize key PCR parameters—particularly the number of cycles and annealing temperature—in order to minimize nonspecific amplification and erroneous incorporation.

These findings not only clarified the main sources of unmapped reads but, more importantly, provided actionable directions for optimizing experimental protocols. Based on these findings, we recommend optimizing PCR conditions (including strict control of cycle numbers), enhancing quality control of oligonucleotide synthesis, and evaluating the fidelity of DNA polymerases to improve library quality. The quality control standards and optimization strategies established in this study have laid an important theoretical foundation for developing more efficient mutant library construction methods.

## Data Availability

All data generated or analyzed during this study are included in this published article and its Supplementary Information Files.

## Supplementary Materials

The following supporting information can be downloaded at: https://www.mdpi.com/article/10.3390/biom15111511/s1, Table S1: The library design and oligo information; Table S2: TAG Mutation Percentages of Various Enzymes under Experimental Conditions; Figure S1: Sequence chromatograms of six isolated clones corresponding to the sequences shown in Figure 3B.

## Appendix A

PCR amplification was performed using the P1 oligonucleotide pool as the template, with the following primers:

PSMD10-amp-F1: 5′-AGCCCGGGGGATCCACCGGTCGCCCATG-3′ and PSMD10-amp-R1: 5′-TCTAGTAGCCAGGGATTTATCGGCCAGAAT-3′.

Platinum SuperFi II DNA Polymerase: The 50 μL PCR reaction mixture contained 1 μL of Platinum SuperFi II DNA Polymerase, 10 μL of 5× SuperFi II Buffer, 1.5 μL of each 10 μM primer, 10 ng of template plasmid, and nuclease-free water to a final volume of 50 μL.

Cycling conditions 1: 1 cycle at 98 °C for 30 s, 14 cycles of 98 °C for 10 s, 60 °C for 10 s, 72 °C for 10 s, followed by final elongation at 72 °C for 5 min.

Cycling conditions 2: 1 cycle at 98 °C for 30 s, 14 cycles of 98 °C for 10 s, 65 °C for 10 s, 72 °C for 10 s, followed by final elongation at 72 °C for 5 min.

Cycling conditions 3: 1 cycle at 98 °C for 30 s, 17 cycles of 98 °C for 10 s, 60 °C for 10 s, 72 °C for 10 s, followed by final elongation at 72 °C for 5 min.

Cycling conditions 4: 1 cycle at 98 °C for 30 s, 17 cycles of 98 °C for 10 s, 65 °C for 10 s, 72 °C for 10 s, followed by final elongation at 72 °C for 5 min.

Q5 Hot Start High-Fidelity DNA Polymerase: The 50 μL PCR reaction mixture contained 25 μL of Q5 Hot Start High-Fidelity DNA Polymerase, 2.5 μL of each 10 μM primer, 10 ng of template plasmid, and nuclease-free water to a final volume of 50 μL.

Cycling conditions 1: 1 cycle at 98 °C for 30 s, 14 cycles of 98 °C for 20 s, 65 °C for 10 s, 72 °C for 10 s, followed by final elongation at 72 °C for 2 min.

Cycling conditions 2: 1 cycle at 98 °C for 30 s, 14 cycles of 98 °C for 20 s, 69 °C for 10 s, 72 °C for 10 s, followed by final elongation at 72 °C for 2 min.

Cycling conditions 3: 1 cycle at 98 °C for 30 s, 17 cycles of 98 °C for 20 s, 65 °C for 10 s, 72 °C for 10 s, followed by final elongation at 72 °C for 2 min.

Cycling conditions 4: 1 cycle at 98 °C for 30 s, 17 cycles of 98 °C for 20 s, 69 °C for 10 s, 72 °C for 10 s, followed by final elongation at 72 °C for 2 min.

Hot-Start Pfu DNA Polymerase: The 50 μL PCR reaction mixture contained 25 μL of Hot-Start Pfu DNA Polymerase, 1.5 μL of each 10 μM primer, 10 ng of template plasmid, and nuclease-free water to a final volume of 50 μL.

Cycling conditions 1: 1 cycle at 95 °C for 3 min, 14 cycles of 95 °C for 30 s, 63 °C for 30 s, 72 °C for 15 s, followed by final elongation at 72 °C for 1 min.

Cycling conditions 2: 1 cycle at 95 °C for 3 min, 14 cycles of 95 °C for 30 s, 65 °C for 30 s, 72 °C for 15 s, followed by final elongation at 72 °C for 1 min.

Cycling conditions 3: 1 cycle at 95 °C for 3 min, 17 cycles of 95 °C for 30 s, 63 °C for 30 s, 72 °C for 15 s, followed by final elongation at 72 °C for 1 min.

Cycling conditions 4: 1 cycle at 95 °C for 3 min, 17 cycles of 95 °C for 30 s, 65 °C for 30 s, 72 °C for 15 s, followed by final elongation at 72 °C for 1 min.

KAPA HiFi HotStart DNA Polymerase: The 50 μL PCR reaction mixture contained 25 μL of KAPA HiFi HotStart DNA Polymerase, 1.5 μL of each 10 μM primer, 10 ng of template plasmid, and nuclease-free water to a final volume of 50 μL.

Cycling conditions 1: 1 cycle at 95 °C for 3 min, 14 cycles of 98 °C for 20 s, 63 °C for 15 s, 72 °C for 10 s, followed by final elongation at 72 °C for 1 min.

Cycling conditions 2: 1 cycle at 95 °C for 3 min, 14 cycles of 98 °C for 20 s, 65 °C for 15 s, 72 °C for 10 s, followed by final elongation at 72 °C for 1 min.

Cycling conditions 3: 1 cycle at 95 °C for 3 min, 17 cycles of 98 °C for 20 s, 63 °C for 15 s, 72 °C for 10 s, followed by final elongation at 72 °C for 1 min.

Cycling conditions 4: 1 cycle at 95 °C for 3 min, 17 cycles of 98 °C for 20 s, 65 °C for 15 s, 72 °C for 10 s, followed by final elongation at 72 °C for 1 min.

TaKaRa Ex Taq DNA Polymerase: The 50 μL PCR reaction mixture contained 0.25 μL of TaKaRa Ex Taq DNA Polymerase, 5 μL of 10× Buffer, 1 μL of 10 μM dNTPs, 1.5 μL of each 10 μM primer, 10 ng of template plasmid, and nuclease-free water to a final volume of 50 μL.

Cycling conditions 1: 14 cycles of 98 °C for 10 s, 60 °C for 30 s, 72 °C for 10 s, followed by final elongation at 72 °C for 1 min.

Cycling conditions 2: 14 cycles of 98 °C for 10 s, 65 °C for 30 s, 72 °C for 10 s, followed by final elongation at 72 °C for 1 min.

Cycling conditions 3: 17 cycles of 98 °C for 10 s, 60 °C for 30 s, 72 °C for 10 s, followed by final elongation at 72 °C for 1 min.

Cycling conditions 4: 17 cycles of 98 °C for 10 s, 65 °C for 30 s, 72 °C for 10 s, followed by final elongation at 72 °C for 1 min.
