# Supplementary figures and images for: A Rapid, High-Throughput Method for the Construction of Mutagenesis Libraries
Source: Biomolecules. 2025 Oct 25;15(11):1511. doi: 10.3390/biom15111511 (PMC12649946; doi:10.3390/biom15111511)

Figure S1

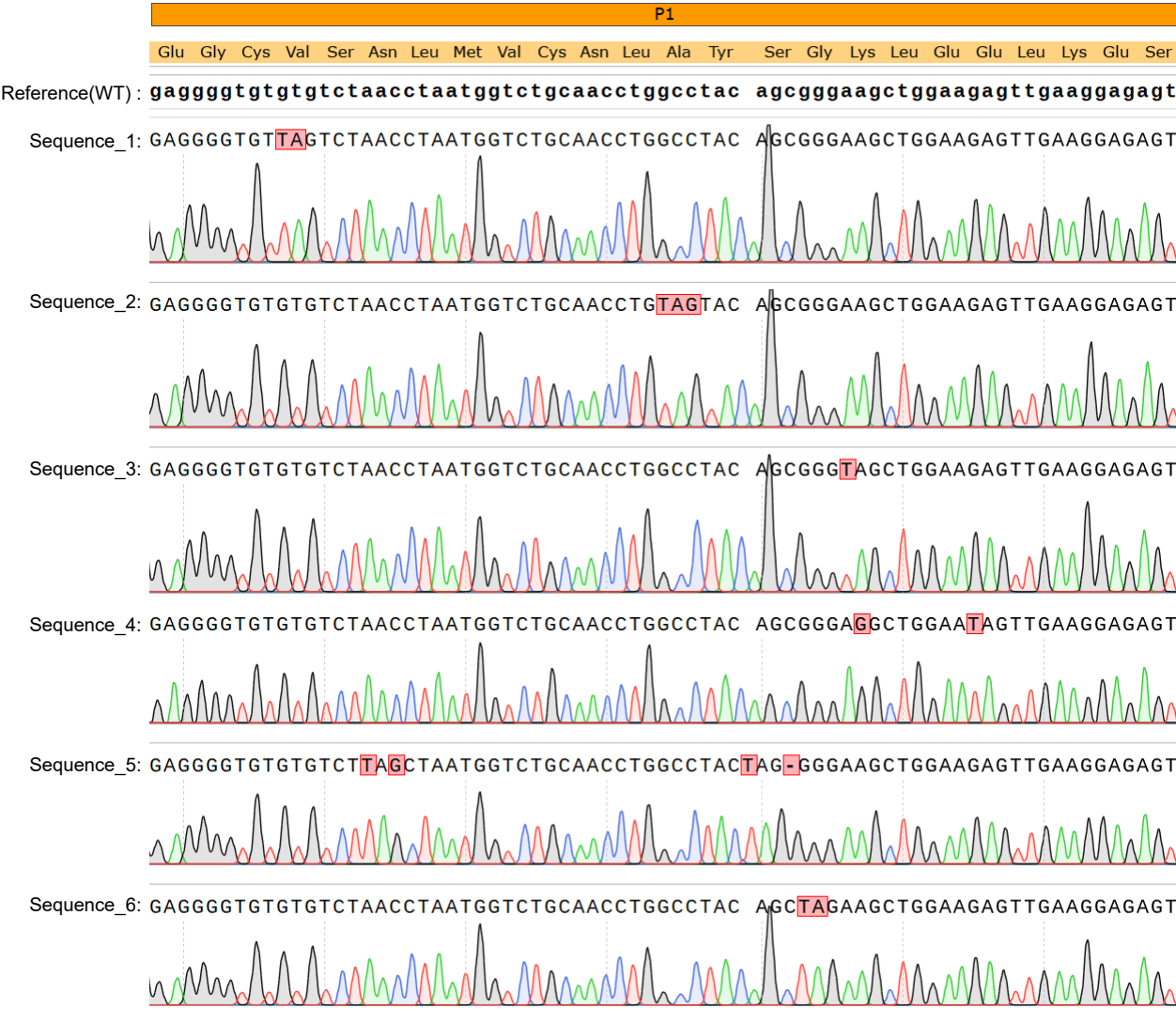

Supplement: Supplementary file 1 [file biomolecules-15-01511-s001.zip › Supplementary Figure S1.pdf]
